# Involvement of inducible nitric oxide synthase and mitochondrial dysfunction in the pathogenesis of enterovirus 71 infection

**DOI:** 10.18632/oncotarget.21250

**Published:** 2017-09-23

**Authors:** Dejian Dang, Chao Zhang, Rongguang Zhang, Weidong Wu, Shuaiyin Chen, Jingchao Ren, Peng Zhang, Guangyuan Zhou, Demin Feng, Tiantian Sun, Ying Li, Qiaoli Liu, Mengchen Li, Yuanlin Xi, Yuefei Jin, Guangcai Duan

**Affiliations:** ^1^ Department of Epidemiology, College of Public Health, Zhengzhou University, Zhengzhou, People's Republic of China; ^2^ Henan Collaborative Innovation Center of Molecular Diagnosis and Laboratory Medicine, Xinxiang, People's Republic of China; ^3^ School of Public Health, Xinxiang Medical University, Xinxiang, People's Republic of China; ^4^ The Third Affiliated Hospital of Zhengzhou University, Zhengzhou, People's Republic of China

**Keywords:** hand foot mouth disease, enterovirus71, inducible nitric oxide synthase, mitochondrial damage, BALB/c mice

## Abstract

Enterovirus71 (EV71) is recognized as the main causative agent of severe hand, foot and mouth disease (HFMD). However, the pathogenesis of EV71 infection has not been well characterized. Clinical evidence indicated that inducible nitric oxide synthase (iNOS) induction in the lung of HFMD patients contributes to the severe symptoms of pulmonary edema. In the present study, we recruited 142 subjects including HFMD patients and controls, and serum level of nitric oxide (NO) was determined. Next, cellular and animal model were used to further investigate the roles of iNOS and mitochondria damage during EV71 infection. Serum NO level in HFMD patients with mild or severe symptoms was higher than that in controls, and there was a trend towards an increase in the serum NO level of severe cases relative to mild cases. EV71 infection caused apoptosis and increased levels of NO, iNOS, superoxide dismutase (SOD) activity and malondialdehyde (MDA), and degraded mitochondrial membrane potential (ΔΨm) *in vitro*. Pathological alterations of mitochondrial morphology were observed *in vitro* and *in vivo*. Furthermore, the expression of iNOS levels in target organs including brain, spinal cord, skeletal muscle, lung and heart were increased with the progression of the pathogenesis of EV71 infection in mice. Taken together, iNOS and mitochondrial damage participate in the pathogenesis of EV71 infection.

## INTRODUCTION

Enterovirus 71 (EV71), belonging to the *Enterovirus* genus *Picornaviridae* family, and coxsackievirus A16 (CA16) are most responsible for hand, foot and mouth disease (HFMD) [1]. Patients infected with EV71, especially infants and preschoolers, are more likely to exhibit severe symptoms comprising aseptic meningitis, brainstem encephalitis, acute flaccid paralysis, myocarditis and pulmonary edema [2]. Although children with mild symptoms can recover in a few days, severe cases usually leave with sequela, and even death. Currently, the increasing incidence of HFMD has been an urgent issue worldwide, especially in Asia-Pacific region [3–7]. It had been reported that about two million children suffered from HFMD, and 129 cases died in mainland China in 2015. Due to restrictions on vaccine and approved antiviral drug for prevention and treatment, it is extremely urgent to reveal the possible mechanisms under the pathogenesis of EV71 infection.

Nitric oxide (NO) is known to be a highly reactive free radical, which acts as a key messenger molecule in regulating series of immune responses [8]. It has been shown that the production of NO is mainly mediated by the nitric oxide synthase (NOS) through modulating the conversion of L-arginine to L-citrulline and NO. NOS has three isoforms including nNOS (neuronal), eNOS (endothelial) and iNOS (inducible). Among them, nNOS and eNOS are endogenously expressed and play critical roles in maintaining normal physiological functions in healthy host whereas many inflammation and infection associated disorders are linked to upregulated iNOS [9–12]. iNOS can be initiated by a variety of extracellular stimulus including lipopolysaccharide (LPS) and cytokines through activation of distinct signaling pathways [13]. Its product, NO, possesses effects of anti-bacteria and anti-virus in innate immune system through mitochondrial damage, and excessive NO acts as a cytotoxic agent in pathological processes, particularly in inflammatory and infectious disorders [9, 14]. EV71 infection was reported to be triggered by comprehensive inflammatory factors [15]. The roles of iNOS and NO in development of cancer [16], muscle injuries [17], and pulmonary injuries [18] have been extensively investigated. Previous study has shown that the iNOS expression in the lungs was greatly increased in EV71 positive cases with pulmonary edema [19]. It has been demonstrated that activated mitochondrial apoptosis pathway was determined in neural and non-neural cells infected with EV71, predicting dysfunction of mitochondria [20]. However the function of iNOS during the pathogenesis of EV71 infection is rarely reported and the mechanism of mitochondrial damage during EV71 infection has not been entirely elucidated yet.

In the present study, the roles of iNOS and mitochondrial damage in the pathogenesis of EV71 infection were investigated, which would help to establish strategies for control and therapy of HFMD.

## RESULTS

### Serum level of NO is increased in severe cases

**Figure 1:**
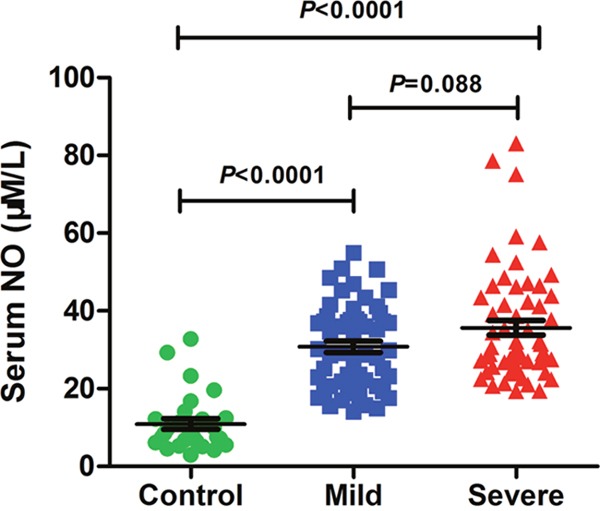
Serum NO level is elevated in severe cases NO level in serum of controls (n=28) and HFMD cases with mild (n=55) or severe symptoms (n=59) was assessed by using related Kit. Data are expressed as means ± SEM.

The average ages of controls and patients with severe and mild symptoms were 18.96 ± 16.22, 19.61 ± 9.11, 20.58 ± 11.98 months, respectively. Three quarters of the controls were males, and they take a proportion of 67.26% and 62.72% in mild or severe cases. There was no significant difference in age and gender among the three groups (*P* > 0.05). Clinical data was summarized in Table 1. No significant difference was found in EV71 positive rate, high fever, heart rate (>130/min), respiratory rate (>35/min) between mild and severe cases. Leukocyte, neutrophil ratio, and C-reaction protein (CRP) in HFMD patients with mild or severe symptoms were significantly higher than those in controls (*P* < 0.05), while lymphocyte ratio, eosinophil ratio and creatinekinase–MB were significantly lower (*P* < 0.05). Nevertheless, no significant difference was found between mild and severe cases (*P* > 0.05). As shown in Figure 1, Serum NO level in mild or severe cases was significantly higher than that in controls (*P* < 0.05). There was a trend towards an increase in the level of serum NO in severe cases relative to mild cases. These results suggest that excessive NO production is involved in HFMD development.

**Table 1:** Characteristics of HFMD patients and controls

Variable	Control(n=28)	Mild(n=55)	Severe(n=59)
EV71 positive, n %	—	5(9.1)	11(18.6)
Fever (>39°C), n %	—	19(34.54)	26(47.27)
Heart rate (>130/min), n %	—	23(41.82)	32(54.24)
Respiratory rate (>35/min), n %	—	5(0.09)	12(0.20)
Leukocytes (×10^9^/L) mean±SD	7.40±1.86	11.65±5.41^b^	10.34±4.68^b^
Neutrophils (%) mean±SD	41.09±13.88	55.00±16.43^a^	57.03±17.14^b^
Lymphocytes (%) mean±SD	49.73±12.41	37.47±16.55^a^	37.04±15.67^b^
Eosinophils (%) mean±SD	2.55±2.46	0.63±0.88^a^	0.45±0.89^b^
C-reaction protein (mg/L) mean±SD	2.02±5.61	26.19±25.84^b^	15.42±27.25^a^
Alanine aminotransferase (U/L) mean±SD	22.42±13.77	18.68±7.01	28±42.93
Aspartate transaminase (U/L) mean±SD	34.41±10.49	32.90±14.56	31.88±7.46
Creatinekinase (U/L) mean±SD	131.86±99.06	124.79±72.88	99.60±79.62
Creatinekinase–MB (U/L) mean±SD	28.15±9.00	18.67±15.61^a^	19.72±17.46^a^

Note: ^a^ *P* < 0.05 vs. control.

^b^ *P* < 0.001 vs. control.

### EV71 infection causes apoptosis in human rhabdomyosarcoma (RD) cells

**Figure 2:**
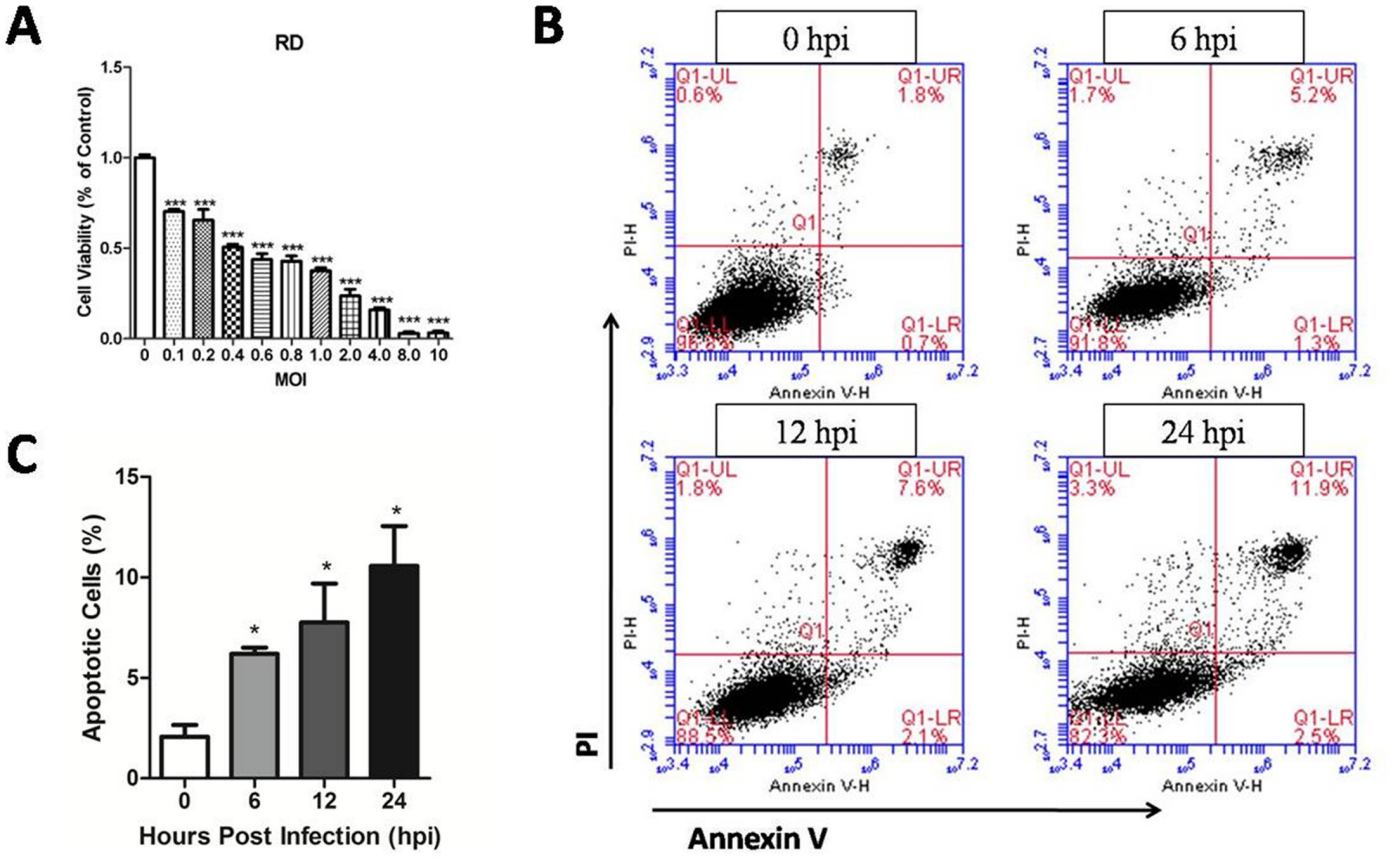
Cytotoxicity of EV71 at different MOI and apoptosis induced by EV71 infection in RD cells **(A)** RD cells were cultured in 96-well plates for 24 h and infected with the EV71 strain for varying MOI. Cell viability was monitored by using a MTT assay kit at 48 hpi. **(B)** RD cell monolayers infected with the EV71 strain at an MOI of 1 were harvested at different time points, and then were stained with PI and Annexin V and examined by Flow Cytometry. **(C)** Apoptotic rate of RD cells at different points post infection. Data are expressed as means ± SEM. *P<0.05, vs 0 hpi (n=3); ***P<0.001, vs 0 MOI (n=3).

EV71 infection can cause RD cells death and the expressions of apoptosis gene [21]. Figure 2A showed that EV71 infection significantly inhibited the cell viability of RD cells with the increasing of multiplicity of infections (MOI) (*P* < 0.05) at 48 hours post infection (hpi). The apoptotic rates of RD cells at 0 hpi, 6 hpi, 12 hpi, and 24 hpi were analyzed by Flow Cytometry using corresponding kit. The apoptotic rates at 6 hpi, 12 hpi, 24 hpi were significantly higher than that at 0 hpi (*P* < 0.05) (Figure 2B and 2C). These results indicate that EV71 infection leads to time-dependent apoptosis in RD cells.

### Endogenous iNOS and NO are involved in EV71 infection-induced cell oxidative damage

**Figure 3:**
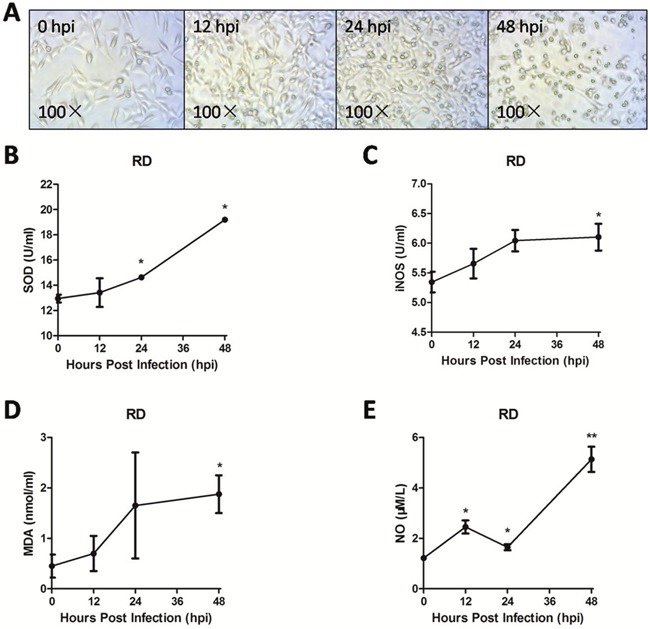
Endogenous iNOS and NO were involved in EV71 infection-induced cell oxidative damage The RD cell monolayers were infected with the EV71 strain of 1 MOI for different periods. The changes of cell morphology **(A)** were observed and captured using a light microscope with amplification (100×) and the levels of SOD activity **(B)**, iNOS **(C)**, MDA **(D)** and NO **(E)** were measured with corresponding assay kit at varying points of infection. Data are expressed as means ± SEM. *P<0.05, vs 0 hpi (n=3); **P<0.01, vs 0 hpi (n=3).

As shown in Figure 3A, RD cells infected with EV71 exhibited significant cytopathic effect (CPE). iNOS, which is responsible for high amounts of NO, can cause cell oxidative damage [13, 22]. The level of iNOS (Figure 3C) in supernatant at 12 hpi, 24 hpi, 48 hpi was higher than that at 0 hpi (*P* < 0.05). In addition, the levels of NO (Figure 3E), superoxide dismutase (SOD) activity (Figure 3B), and malondialdehyde (MDA) (Figure 3D) in supernatant at 48 hpi were all significantly higher than that at 0 hpi (*P* < 0.05). These results suggest that the increase of endogenous iNOS and NO are involved in EV71 infection-induced cell damage *in vitro*.

### EV71 infection induces mitochondrial damage *in vivo* and *in vitro*

**Figure 4:**
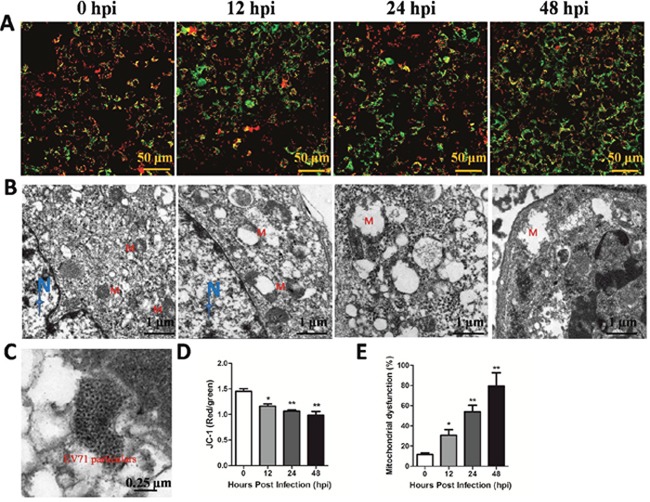
EV71 infection induced mitochondrial damage *in vitro* RD cells (5×10^5^) cultured in confocal dish for 24 h were infected with EV71 and harvested at different time points. **(A)** JC-1 probe was used to investigate ΔΨm after EV71 infection in RD cells under a confocal microscopy (bar=50 μm). **(B)** The ultramicro variation of organellae in infected RD cells under TEM (bar=1 μm). N and M represents nucleus and mitochondria, respectively. **(C)** The location of EV71 particles in infected RD cells (bar=0.25 μm). **(D)** The red/green rate representing ΔΨm was evaluated by Image-Pro Plus 6.0 software. **(E)** Mitochondrial dysfunction (%) in RD cells after EV71 infection at different time points. Data are expressed as means ± SEM. *P<0.05, vs 0 hpi (n=3); **P<0.01, vs 0 hpi (n=3).

**Figure 5:**
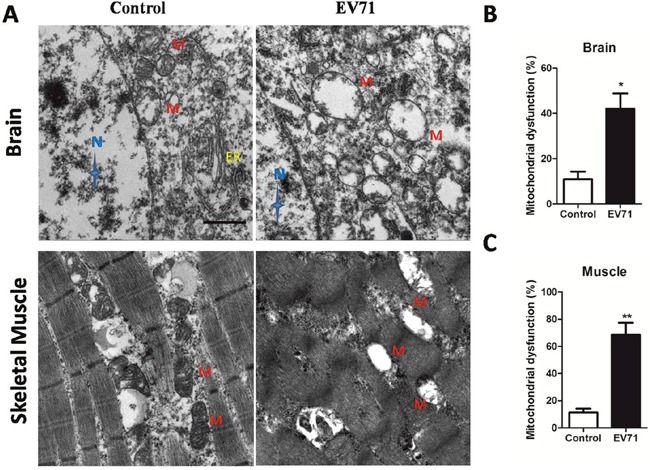
EV71 infection induced mitochondrial damage in structural cells of brain and skeletal muscle from mice **(A)** Mitochondrial dysfunction in brain and skeletal muscle from mice with EV71 infection at 7 dpi was observed under TEM (bar=1 μm). N, M and ER represent nucleus, mitochondria and endoplasmic reticulum, respectively. Mitochondrial dysfunction (%) in brain cells **(B)** and skeletal muscle cells **(C)** was calculated by the number of damaged mitochondria in the total number of mitochondria under three different versions. Data are expressed as means ± SEM. *P<0.05, vs 0 hpi (n=4); **P<0.01, vs Control (n=4).

Endogenous iNOS is known to be linked with mitochondrial damage which can trigger anti-virus effects in innate immune [9, 14]. In the present study, TEM or JC-1 probe was used to investigate mitochondrial dysfunction after EV71 infection *in vitro* and *in vivo* model. As shown in Figure 4A and 4D, EV71 infection reduced the ratios of red/green at 12 hpi, 24 hpi and 48 hpi, representing degraded mitochondrial membrane potential (ΔΨm) in RD cells with a time-dependent trend (*P* < 0.05). Additionally, as shown in Figure 4B, the mitochondrial morphology of infected RD cells at 12 hpi under TEM showed pathological changes including enlarged external membrane and mitochondrial ridge reduction compared to normal organelle at 0 hpi. The swelling of mitochondria and mitochondrial ridge reduction were more obvious at 24 hpi. At 48 hpi the mitochondria of infected RD cells were moved to cytomembrance through ruptured membrane. EV71 particles were also found in infected RD cells (Figure 4C). The rate of mitochondrial dysfunction (%) was applied to evaluate the degree of mitochondrial damage. As shown in Figure 4E, mitochondrial dysfunction (%) of infected RD cells at 12 hpi, 24 hpi and 48 hpi was higher than that in control cells (*P*<0.05). As shown in Figure 5A, the mitochondrial damage was also found in neuron and skeletal muscle cells from infected mice, and the quantitative results of mitochondrial dysfunction (%) in brain (Figure 5B) and skeletal muscle (Figure 5C) were enhanced after EV71 infection (*P*<0.05). Our results suggest that EV71 infection leads to pronounced mitochondrial dysfunction *in vitro* and *in vivo*.

### iNOS involvement in the development of EV71 infection in mice

**Figure 6:**
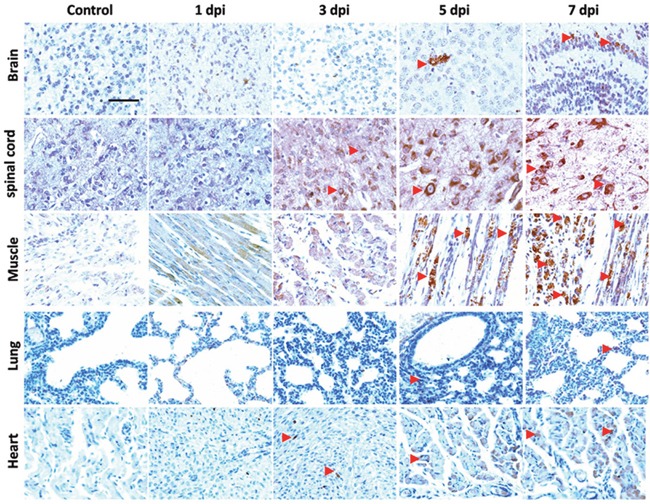
Localization of EV71 VP1 in tissues of EV71-infected mice Three days old BALB/c mice were intraperitoneally incubated with EV71 (2×10^6^ PFU) and were sacrificed at 1 dpi, 3 dpi, 5 dpi, 7 dpi. Slices of brain, spinal cord, skeletal muscle, lung and heart of mice were stained with anti-EV71 VP1 antibody. The red triangle indicates the positive staining (brown). Bar=50 μm.

**Figure 7:**
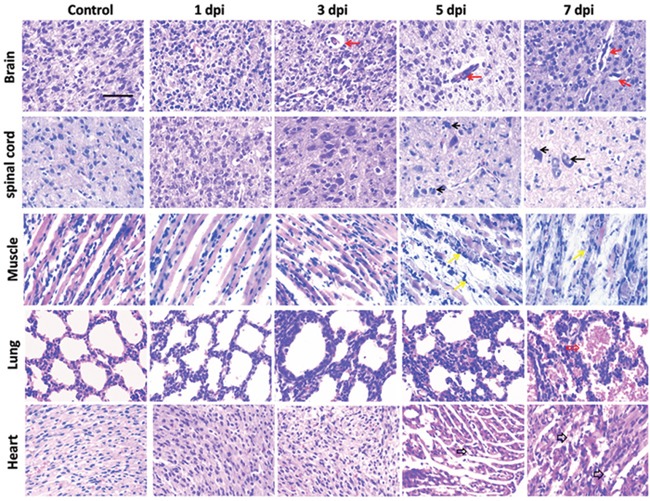
Histopathological examinations of tissues in mice after EV71 infection Tissues dissected out from EV71-infected 3-day-old mice at different time points post infection were sliced and stained with H&E. Brain tissues exhibited perivascular cuffing and neuronal degeneration (indicated by red solid arrows). Hyperchromatic and pyknotic nerve cells, neuronal loss and glial nodules (indicated by black solid arrows) were found in spinal cord. Cardiac muscle and skeletal muscle appeared necrotizing myositis with muscle fibers rupture and inflammatory cell infiltration (indicated by black hollow arrows and yellow arrows, respectively) at day 5 and 7 post infection. Lung comprised swollen alveolus pulmonis at 5 dpi and erythrocyte-filled fluid in the alveolar spaces (indicated by red hollow arrows) at 7 dpi. Bar= 50 μm.

Immunohistochemical (IHC) staining of EV71 VP1 was applied to track the invasion of EV71 *in vivo*. As shown in Figure 6, IHC positive staining of VP1 (brown) was detected in brain, spinal cord, skeletal muscle, lung and heart from mice with EV71 infection at 3 days post infection (dpi), 5 dpi or 7 dpi. However, there was no signal in healthy controls. Furthermore, histopathological alterations of mice at 1, 3, 5, 7 dpi were presented with haematoxylin and eosin (H&E) staining. As shown in Figure 7, brain tissues from infected mice exhibited pathological changes including perivascular cuffing and neuronal degeneration compared to healthy controls. Pyknotic nerve cells, neuronal loss and glial nodules were found in spinal cord of infected mice. Cardiac muscle and skeletal muscle appeared necrotizing myositis with muscle fibers rupture and inflammatory cells infiltration at 5 dpi and 7 dpi. Severe lesions such as swollen alveoli and erythrocyte-filled fluid in the alveolar spaces were detected in lungs of infected mice at 5 or 7 dpi. The above results indicate that EV71 infection induces obvious histopathological alterations in target organs.

**Figure 8:**
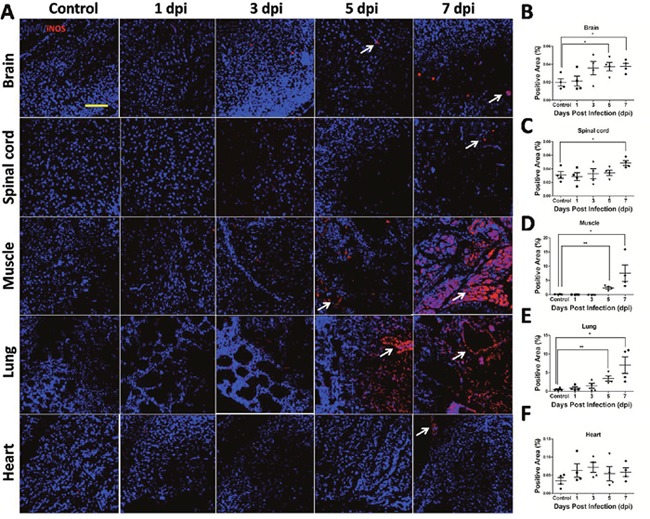
iNOS expression in target organs of EV71-infected mice **(A)** iNOS expression level in target organs from mice with EV71 infection was evaluated by immunofluorescence under confocal microscopy (bar=50 μm). White arrows showed positive staining (red). Positive areas (%) of iNOS expression in the cytoplasm of brain **(B)**, spinal cord **(C)**, skeletal muscle **(D)**, lung **(E)** and heart **(F)** were estimated by Image-Pro Plus 6.0 software. Data are expressed as means ± SEM. *P<0.05, vs 0 hpi (n=4); **P<0.01, vs Control (n=4).

In order to determine the involvement of iNOS in EV71 infected mice, expression level of iNOS in brain, spinal cord, skeletal muscle, lung and heart was analyzed with immunofluorescence method. As shown in Figure 8A, the increase in positive staining of iNOS (red) was observed in brain, spinal cord, skeletal muscle, lung and heart after EV71 infection, especially in lung, skeletal muscle at 5 dpi and 7 dpi. Quantitative results showed increased expression of iNOS in brain (Figure 8B), skeletal muscle (Figure 8D), lung (Figure 8E) at 5 dpi and 7 dpi, and in spinal cord (Figure 8C) at 7 dpi compared to controls (*P* < 0.05). Taken together, these results suggest that iNOS is involved in the pathogenesis of EV71 infection.

## DISCUSSION

Recently, although pathogen spectrum of HFMD has changed, EV71 is still a main pathogen and responsible for most death cases [23–25]. Thus, it is extremely urgent to clarify the underlying mechanisms of EV71 infection. In this study, we found serum NO level of HFMD patients was pronouncedly increased, and higher level was detected in severe cases, although there was no significant difference (with a p value of 0.088) relative to mild. Then, RD cells and 3-day-old BALB/c mice were infected with an EV71 strain to investigate the possible mechanisms of iNOS in the pathogenesis of EV71 infection. We found EV71 infection induced RD cells apoptosis, and elevated levels of iNOS, NO, SOD activity, and MDA were detected in the supernatant of infected RD cells with impaired mitochondrial function. Mitochondria in brain and skeletal muscle structural cells exhibited similar alterations. Additionally, we observed an excessive expression of iNOS in target organs of infected mice accompanied by histopathological alterations.

Previous study highlighted a great increment of iNOS in lungs from an EV71-infected fatal case with acute respiratory distress syndrome. In support of this observation, our results demonstrated an elevated NO level in serum of HFMD cases. What's more, serum NO level in severe cases showed a trend towards an increase compared to mild cases. We also found that serum interleukin (IL)-6 level in severe patients were higher than mild patients (data not show), which had been reported in many studies [15, 26, 27]. Hence, we assumed that iNOS may be involved in the development of HFMD.

It has been well established that physiological levels of NO act as part of the host defense against pathogens, whereas excessive iNOS and NO production may be harmful [28]. iNOS, existing in a number of cells, such as endothelium, hepatocytes, monocytes, mast cells, macrophages and smooth muscle cells, catalyzes NO production under the stimulation of bacteria LPS and cytokines such as interferon-α (IFN-α), IL-1β, IL-6, tumor necrosis factor-α (TNF-α) or other compounds [14, 29]. Cascaded cytokines production was verified in HFMD patients with EV71 infection, especially those combined with central nervous system symptom, brainstem encephalitis and pulmonary edema [15, 27, 30]. Taken together, our findings indicate that iNOS may play a critical role in the development of HFMD. For further study, cellular and animal model were used to identify the role of iNOS.

The released NO in the microenvironment is known to initiate the apoptotic pathway [31]. In the present study, we found that EV71 infection induced apoptosis and inhibited viability of RD cells. Overproduction of NO is known to attack mitochondria, cell membranes, DNA, and proteins, resulting in oxidative damage to cells or tissues [32]. Our study found elevated activation of SOD, an important antioxidant, and increased MDA, which represents lipid peroxidation. In combination, these results suggested that EV71 infection induces oxidative stress in infected RD cells. It has been reported that NO possesses effects of anti-bacteria and anti-virus in innate immune system through mitochondrial damage [9, 14]. In the current study, decreased ΔΨm, a biomarker of the prophase of apoptosis and swelled mitochondria containing large dilations were detected in RD cells. Similar changes appeared in structural cells in brain and skeletal muscle, indicating EV71 infection can induce mitochondrial dysfunction *in vitro* and *in vivo*. Previous studies have also demonstrated that mitochondrial dysfunction plays a critical role in the development of virus infection under oxidative stress [33, 34].

Therefore, we established an animal model to investigate the possible mechanism of iNOS in EV71 infection. Interestingly, increasing expression level of iNOS was found in the brain, spinal cord, skeletal muscle and lung from mice with EV71 infection. Previous study indicated that EV71 infection caused neuronal cells death by activation of glia with an elevation of NO [35]. It has been acknowledged that iNOS induction mediates neuronal inflammation and neuronal injury [36]. In this study, EV71 invasion and perivascular cuffing, neuronal degeneration and glial nodules were observed in brain and spinal cord. iNOS may be an important mediator, leading to central nervous system (CNS) injury after EV71 infection. Severe injuries were observed in skeletal muscle with appearance of a large quantity of viral antigens in the present study. Skeletal muscle inflammation and damage have been thought to be one of the main reasons why clinical severe cases exhibit symptom of limb paralysis [37]. So far, little is known about the mechanisms of muscle lesions caused by EV71 infection. In this study, a massive expression of iNOS was found in skeletal muscle. It has been indicated that iNOS expression can be greatly increased in skeletal muscle cells after exposure to inflammatory cytokines and lead to rat skeletal muscle myoblasts apoptosis [38, 39]. Considering the inflammatory cells infiltration appeared in skeletal muscle, we postulate that activated iNOS and its derivatives might contribute to skeletal muscle injury. Importantly, we also found large amount of iNOS expression in mouse lungs after EV71 infection. iNOS has been reported to play an essential role in the development of pulmonary edema induced by intestinal ischemia-reperfusion (I/R) and lung injury caused by ventilator [40, 41]. Moreover, NO can mediate lung vascular permeability [42]. The substantial iNOS accompanied by pulmonary edema in mice with EV71 infection supports the clinical notion that the iNOS induction in the lungs contributes to severe symptom with pulmonary edema in EV71 positive cases [19].

## MATERIALS AND METHODS

### Ethical statement

Approvals were authorized by the Life Sciences and Ethics Committee of Zhengzhou University and the Ethics Committee of the Children's Hospital of Zhengzhou. Written informed consent was obtained from participants or their guardians.

### Subjects

HFMD patients diagnosed following “Hand, foot and mouth disease treatment guidelines” (Chinese Ministry of Public Health, revised in 2010) in the Children's Hospital of Zhengzhou from April 2015 to November 2015 were recruited. Encephalitis, acute flaccid paralysis, myocarditis and pulmonary edema were classified as severe symptoms [43]. Children with inguinal hernia treated in The Third Affiliated Hospital of Zhengzhou University from January 2016 to December 2016 were enrolled as controls. A total of 142 subjects including 55 mild cases, 59 severe cases and 28 controls without HFMD were divided into three different groups. Serum of HFMD cases and controls was collected and stored at −80°C until determination. Clinical data was recorded and shown in Table 1.

### Determination of serum NO

Serum NO level from HFMD cases and controls was detected by Griess method, as indicated on the NO assay kit (Beyotime Biotech Inc., Jiangsu, China).

### Cells and EV71 virus

Human RD cells were maintained in Dulbecco's modified Eagle's medium (DMEM, Gibco Company, New York, USA) supplemented with 10% fetal bovine serum (FBS, Gibco Company, New York, USA) at 37°C in a 5% CO_2_ humidified incubator. The EV71 strain isolated from a hospitalized child combined CNS symptoms in Children's Hospital of Zhengzhou (Zhengzhou, Henan, China) was grown in RD cells. Stocks used in this study were 10^8^ PFU per ml.

### Animals

BALB/c mice (SPF degree) were purchased from the Medical Animal Center in Zhengzhou University, Henan, China, and raised in individual ventilation cage (IVC) system. Sixteen 3-day-old BALB/c mice (n=4 for each group) were inoculated intraperitoneally with EV71 strain (2×10^6^ PFU) and sacrificed with isoflurane on 1, 3, 5 and 7 days post infection (dpi). The 3-day-old mice (n=4) injected with the same volume of RD cell culture supernatants and sacrificed with isoflurane on 7 dpi were used as controls.

### Cytotoxicity of EV71 strain

RD cells were seeded in 96-well plates (2×10^4^ cells/well) with 200 μL DMEM medium supplemented with 10% FBS for 24 h and infected with the EV71 strain at a MOI of 0.1, 0.2, 0.4, 0.8, 1.0, 2.0, 4.0, 8.0, and 10. Cell viability was monitored by a MTT assay kit (Vazyme Biotech Co.Ltd., Nanjing, China) at 48 hpi according to the manufacturers’ instruction.

2×10^5^ cells RD cells were seeded in 24-well plates with 1 mL DMEM medium supplemented with 10% FBS. The apoptosis of RD cells after 1 MOI EV71 infection at 0 hpi, 6 hpi, 12 hpi and 24 hpi was measured by Flow Cytometry (BD Immunocytometry Systems, San Jose, Calif.) using Annexin V-FITC Apoptosis Detection Kit (Vazyme Biotech Co.Ltd., Nanjing, China). Annexin V or double positive cells were apoptotic cells.

### Analysis of culture supernatant

RD cells seeded in 6-well plates (10^6^ cells/well) with 500ml DMEM medium supplemented with 10% FBS were infected with EV71 strain at 1 MOI and the supernatants were harvested at 0 hpi, 12 hpi, 24 hpi, 48 hpi. Levels of SOD activity, MDA, iNOS in supernatant were measured using relative kits purchased from Nanjing Jiancheng Bioengineering Institute (Nanjing, China). NO was determined by the Griess method, as indicated in NO assay kit (Beyotime Biotech Inc., Jiangsu, China). All the experimental procedures were conducted strictly following the manufacturer's instructions.

### Histopathology and immunohistochemistry

Mice were sacrificed on 1, 3, 5, 7 dpi, and brain, spinal cord, lung, skeletal muscle, heart of mice were immediately fixed in 4% paraformaldehyde at 4°C overnight. After fixation, paraffin-embedded tissues of 5 μm in thickness were stained with H&E. The viral VP1 antigens were detected and localized by IHC staining. Briefly, the paraffin section slides were incubated with 0.5% H_2_O_2_ for 10 min and washed three times with phosphate buffer saline (PBS) after microwaved, and then blocked in a 5% bovine serum albumin (BSA) solution for 30 min. Rabbit anti-EV71 VP1 polyclonal antibodies (GeneTex, Inc, San Antonio, USA 1:500 dilution) were used to incubate infected sections at 4°C overnight. The tissue sections were washed three times with PBS and then incubated with horse radish peroxidase (HRP) -conjugated anti-rabbit secondary antibody (1:3000 dilution) for 30 min at 37°C. The sections developed with 3-3′ diaminobenzidine (DAB) and counterstained with hematoxylin were viewed with a light microscope.

### Transmission electron microscopy (TEM)

RD cell monolayers in 6-well plates (10^6^ cells/well) were harvested after infection with 1MOI EV71 at 0 hpi, 12 hpi, 24 hpi and 48 hpi, and were washed with 1×PBS. The cell pellets were fixed with 2.5% glutaraldehyde in 0.1 M sodium cacodylate buffer for 1.5 h at 4°C, post-fixed in cacodylate buffer containing 1% OsO_4_ for 1 h at 4°C. The samples were then stained with 1% uranyl acetate buffer overnight at 4°C in the process of 80% ethanol dehydration during dewatered with gradient ethanol. The cells were embedded in ethoxyline resin (Embed812EMS) and sectioned at a thickness of 60 nm. The sections collected on formver/carbon-coated grids were stained with 2% uranyl acetate and lead citrate buffer for 30 min and observed under an FEI Tecnai G2 Spirit Biotwin TEM at 200 kV. Pictures were captured by Morada CCD and iTEM software (Olympus Optical Co.Ltd., Tokyo, Japan). The brain and skeletal muscle of mice without or with EV71 infection at 7 dpi were sliced into 1 mm^3^ cube with a sharp blade. The samples were handled as described above.

The rate of mitochondrial dysfunction (%) was calculated by the number of damaged mitochondria in the total number of mitochondria under three different versions.

### Immunofluorescence

The ΔΨm of infected RD cells was assessed by an assay kit with JC-1 probe (Beyotime biotech Co.Ltd, Shanghai, China). In detail, 5×10^5^ RD cells were seeded in confocal dish with 2 mL DMEM containing 10% FBS for 24 h, and then were infected with 1 MOI EV71. Infected RD cells were harvested at 0 hpi, 12 hpi, 24 hpi and 48 hpi, and washed twice with 1×PBS. The infected cells were incubated for 20 min at 37°C with 1 ml JC-1 fluid and 1 ml DMEM. After that, the cell monolayers were gently washed twice with JC-1 buffer and then 2 ml DMEM were added. Pictures were captured under a Leica TCS-SP8 confocal microscopy (Leica Microsystem, Wetzlar, Germany). The red/green rate representing ΔΨm was evaluated by Image-Pro Plus 6.0 software.

Paraffin-embedded tissues of 5 μm in thickness were dewaxed and washed three times with 1×PBS. 1×PBS containing 0.5% Triton X-100 was applied to permeabilize sections, and then the sections were blocked for 30 min with goat serum at room temperature. Rabbit anti-mouse iNOS (EnoGene Biotechnology Co., Ltd., Nanjing, China 1:100 dilution) was added and incubated overnight at 4°C, followed by incubation with Cy3-conjugated goat anti-rabbit secondary antibodies at a concentration of 1:1000 at 37°C for 30 min. The images were captured using Leica TCS-SP8 confocal microscopy after stained with DAPI for 5 min. Positive area of iNOS expression was reflected as the red staining in the cytoplasm and estimated by Image-Pro Plus 6.0 software.

### Statistical analysis

Data was presented as mean ± SEM. SPSS21.0 (IBM, NC, USA) was used for statistical analysis. Data comparison in multiple groups was carried out by one-way ANOVAs, and the further comparisons between two groups were followed by Dunnett's post-test or adjusted by Bonferroni method. Two-tailed Student's t-test or Mann-Whitney U test was performed in comparison of two groups according to the distribution of data. A *P* value of <0.05 was considered statistically significant.

## CONCLUSIONS

Our study first indicated that the involvement of iNOS and mitochondrial dysfunction were critical events in the development of EV71 infection. In the near future, iNOS inhibitor is required to further uncover the mechanisms of EV71 infection associated HFMD.

## References

[1] Ho M, Chen ER, Hsu KH, Twu SJ, Chen KT, Tsai SF, Wang JR, Shih SR (1999). An epidemic of enterovirus 71 infection in Taiwan. Taiwan Enterovirus Epidemic Working Group. N Engl J Med.

[2] Chang LY, Lin TY, Hsu KH, Huang YC, Lin KL, Hsueh C, Shih SR, Ning HC, Hwang MS, Wang HS, Lee CY (1999). Clinical features and risk factors of pulmonary oedema after enterovirus-71-related hand, foot, and mouth disease. Lancet.

[3] Tan X, Huang X, Zhu S, Chen H, Yu Q, Wang H, Huo X, Zhou J, Wu Y, Yan D, Zhang Y, Wang D, Cui A (2011). The persistent circulation of enterovirus 71 in People's Republic of China: causing emerging nationwide epidemics since 2008. PLoS One.

[4] Chia MY, Chiang PS, Chung WY, Luo ST, Lee MS (2014). Epidemiology of enterovirus 71 infections in Taiwan. Pediatr Neonatol.

[5] Kim HJ, Hyeon JY, Hwang S, Lee YP, Lee SW, Yoo JS, Kang B, Ahn JB, Jeong YS, Lee JW (2016). Epidemiology and virologic investigation of human enterovirus 71 infection in the Republic of Korea from 2007 to 2012: a nationwide cross-sectional study. BMC Infect Dis.

[6] Ang LW, Koh BK, Chan KP, Chua LT, James L, Goh KT (2009). Epidemiology and control of hand, foot and mouth disease in Singapore, 2001-2007. Ann Acad Med Singapore.

[7] Fujimoto T, Chikahira M, Yoshida S, Ebira H, Hasegawa A, Totsuka A, Nishio O (2002). Outbreak of central nervous system disease associated with hand, foot, and mouth disease in Japan during the summer of 2000: detection and molecular epidemiology of enterovirus 71. Microbiol Immunol.

[8] Hibbs JB, Taintor RR, Vavrin Z, Rachlin EM (1988). Nitric oxide: a cytotoxic activated macrophage effector molecule. Biochem Biophys Res Commun.

[9] Burggraaf S, Bingham J, Payne J, Kimpton WG, Lowenthal JW, Bean AG (2011). Increased inducible nitric oxide synthase expression in organs is associated with a higher severity of H5N1 influenza virus infection. PLoS One.

[10] Avdagic N, Zaciragic A, Babic N, Hukic M, Seremet M, Lepara O, Nakas-Icindic E (2013). Nitric oxide as a potential biomarker in inflammatory bowel disease. Bosn J Basic Med Sci.

[11] Sioutas A, Ehren I, Lundberg JO, Wiklund NP, Gemzell-Danielsson K (2008). Intrauterine nitric oxide in pelvic inflammatory disease. Fertil Steril.

[12] MacMicking J, Xie QW, Nathan C (1997). Nitric oxide and macrophage function. Annu Rev Immunol.

[13] Lowenstein CJ, Padalko E (2004). iNOS (NOS2) at a glance. J Cell Sci.

[14] Aktan F (2004). iNOS-mediated nitric oxide production and its regulation. Life Sci.

[15] Ye N, Gong X, Pang LL, Gao WJ, Zhang YT, Li XL, Liu N, Li DD, Jin Y, Duan ZJ (2015). Cytokine responses and correlations thereof with clinical profiles in children with enterovirus 71 infections. BMC Infect Dis.

[16] Vannini F, Kashfi K, Nath N (2015). The dual role of iNOS in cancer. Redox Biol.

[17] Kim K (2015). Interaction between HSP 70 and iNOS in skeletal muscle injury and repair. J Exerc Rehabil.

[18] Mehta S (2005). The effects of nitric oxide in acute lung injury. Vascul Pharmacol.

[19] Kao SJ, Yang FL, Hsu YH, Chen HI (2004). Mechanism of fulminant pulmonary edema caused by enterovirus 71. Clin Infect Dis.

[20] Chang SC, Lin JY, Lo LY, Li ML, Shih SR (2004). Diverse apoptotic pathways in enterovirus 71-infected cells. J Neurovirol.

[21] Shi W, Li X, Hou X, Peng H, Jiang Q, Shi M, Ji Y, Liu X, Liu J (2012). Differential apoptosis gene expressions of rhabdomyosarcoma cells in response to enterovirus 71 infection. BMC Infect Dis.

[22] Michel T, Feron O (1997). Nitric oxide synthases: which, where, how, and why?. J Clin Invest.

[23] Zhuang ZC, Kou ZQ, Bai YJ, Cong X, Wang LH, Li C, Zhao L, Yu XJ, Wang ZY, Wen HL (2015). Epidemiological Research on Hand, Foot, and Mouth Disease in Mainland China. Viruses.

[24] Liu SL, Pan H, Liu P, Amer S, Chan TC, Zhan J, Huo X, Liu Y, Teng Z, Wang L, Zhuang H (2015). Comparative epidemiology and virology of fatal and nonfatal cases of hand, foot and mouth disease in mainland China from 2008 to 2014. Rev Med Virol.

[25] Xu W, Liu CF, Yan L, Li JJ, Wang LJ, Qi Y, Cheng RB, Xiong XY (2012). Distribution of enteroviruses in hospitalized children with hand, foot and mouth disease and relationship between pathogens and nervous system complications. Virol J.

[26] Chen ZF, Li RQ, Xie ZC, Huang GQ, Yuan QC, Zeng JC (2014). IL-6, IL-10 and IL-13 are associated with pathogenesis in children with Enterovirus 71 infection. International Journal Of Clinical And Experimental Medicine.

[27] Lin TY, Hsia SH, Huang YC, Wu CT, Chang LY (2003). Proinflammatory cytokine reactions in enterovirus 71 infections of the central nervous system. Clin Infect Dis.

[28] Bogdan C, Rollinghoff M, Diefenbach A (2000). Reactive oxygen and reactive nitrogen intermediates in innate and specific immunity. Curr Opin Immunol.

[29] Kleinert H, Pautz A, Linker K, Schwarz PM (2004). Regulation of the expression of inducible nitric oxide synthase. Eur J Pharmacol.

[30] Wang SM, Lei HY, Huang KJ, Wu JM, Wang JR, Yu CK, Su IJ, Liu CC (2003). Pathogenesis of enterovirus 71 brainstem encephalitis in pediatric patients: roles of cytokines and cellular immune activation in patients with pulmonary edema. J Infect Dis.

[31] Mula RV, Machiah D, Holland L, Wang X, Parihar H, Sharma AC, Selvaraj P, Shashidharamurthy R (2016). Immune Complex-Induced, Nitric Oxide-Mediated Vascular Endothelial Cell Death by Phagocytes Is Prevented with Decoy FcgammaReceptors. PLoS One.

[32] Liu X, Guo P, Liu A, Wu Q, Xue X, Dai M, Hao H, Qu W, Xie S, Wang X, Yuan Z (2017). Nitric oxide (NO)-mediated mitochondrial damage plays a critical role in T-2 toxin-induced apoptosis and growth hormone deficiency in rat anterior pituitary GH3 cells. Food Chem Toxicol.

[33] Apostolova N, Funes HA, Blas-Garcia A, Alegre F, Polo M, Esplugues JV (2015). Involvement of nitric oxide in the mitochondrial action of efavirenz: a differential effect on neurons and glial cells. J Infect Dis.

[34] Kammouni W, Wood H, Saleh A, Appolinario CM, Fernyhough P, Jackson AC (2015). Rabies virus phosphoprotein interacts with mitochondrial Complex I and induces mitochondrial dysfunction and oxidative stress. J Neurovirol.

[35] Chang CY, Li JR, Ou YC, Chen WY, Liao SL, Raung SL, Hsiao AL, Chen CJ (2015). Enterovirus 71 infection caused neuronal cell death and cytokine expression in cultured rat neural cells. Iubmb Life.

[36] Zhu Y, Jones G, Tsutsui S, Opii W, Liu S, Silva C, Butterfield DA, Power C (2005). Lentivirus infection causes neuroinflammation and neuronal injury in dorsal root ganglia: pathogenic effects of STAT-1 and inducible nitric oxide synthase. J Immunol.

[37] Lin P, Gao L, Huang Y, Chen Q, Shen H (2015). An enterovirus 71 strain causes skeletal muscle damage in infected mice. Int J Clin Exp Pathol.

[38] Stamler JS, Meissner G (2001). Physiology of nitric oxide in skeletal muscle. Physiol Rev.

[39] Stangel M, Zettl UK, Mix E, Zielasek J, Toyka KV, Hartung HP, Gold R (1996). H2O2 and nitric oxide-mediated oxidative stress induce apoptosis in rat skeletal muscle myoblasts. J Neuropathol Exp Neurol.

[40] Turnage RH, Wright JK, Iglesias J, LaNoue JL, Nguyen H, Kim L, Myers S (1998). Intestinal reperfusion-induced pulmonary edema is related to increased pulmonary inducible nitric oxide synthase activity. Surgery.

[41] Liu R, Hotta Y, Graveline AR, Evgenov OV, Buys ES, Bloch KD, Ichinose F, Zapol WM (2007). Congenital NOS2 deficiency prevents impairment of hypoxic pulmonary vasoconstriction in murine ventilator-induced lung injury. Am J Physiol Lung Cell Mol Physiol.

[42] Breithaupt-Faloppa AC, Vitoretti LB, Coelho FR, dos Santos Franco AL, Domingos HV, Sudo-Hayashi LS, Oliveira-Filho RM, Tavares de Lima W (2009). Nitric oxide mediates lung vascular permeability and lymph-borne IL-6 after an intestinal ischemic insult. Shock.

[43] Duan G, Yang H, Shi L, Sun W, Sui M, Zhang R, Wang X, Wang F, Zhang W, Xi Y, Fan Q (2014). Serum inflammatory cytokine levels correlate with hand-foot-mouth disease severity: a nested serial case-control study. PLoS One.

